# Perturbations of MicroRNA Function in Mouse Dicer Mutants Produce Retinal Defects and Lead to Aberrant Axon Pathfinding at the Optic Chiasm

**DOI:** 10.1371/journal.pone.0010021

**Published:** 2010-04-01

**Authors:** Rita Pinter, Robert Hindges

**Affiliations:** MRC Centre for Developmental Neurobiology, King's College London, Guy's Campus, London, United Kingdom; Institut de la Vision, France

## Abstract

**Background:**

During development axons encounter a variety of choice points where they have to make appropriate pathfinding decisions. The optic chiasm is a major decision point for retinal ganglion cell (RGC) axons en route to their target in order to ensure the correct wiring of the visual system. MicroRNAs (miRNAs) belong to the class of small non-coding RNA molecules and have been identified as important regulators of a variety of processes during embryonic development. However, their involvement in axon guidance decisions is less clear.

**Methodology/Principal Findings:**

We report here that the early loss of Dicer, an essential protein for the maturation of miRNAs, in all cells of the forming retina and optic chiasm leads to severe phenotypes of RGC axon pathfinding at the midline. Using a conditional deletion approach in mice, we find in homozygous *Dicer* mutants a marked increase of ipsilateral projections, RGC axons extending outside the optic chiasm, the formation of a secondary optic tract and a substantial number of RGC axons projecting aberrantly into the contralateral eye. In addition, the mutant mice display a microphthalmia phenotype.

**Conclusions:**

Our work demonstrates an important role of Dicer controlling the extension of RGC axons to the brain proper. It indicates that miRNAs are essential regulatory elements for mechanisms that ensure correct axon guidance decisions at the midline and thus have a central function in the establishment of circuitry during the development of the nervous system.

## Introduction

During development the correct patterning of tissues and the action of a network of guidance and signaling proteins ensure the accurate establishment of neural connectivity. The visual system is a well-studied model system to investigate these fundamental processes. Retinal ganglion cells (RGCs) are the only cells sending out axons from the eye and transmit information to a number of targets in the brain. The hierarchical interplay between different transcription factors ensures that the appropriate axon guidance molecules are expressed within RGC axons and in the target tissues [1]. For example Tbx5 [2] and Vax2 [3] are transcription factors that specify the dorsal (D) and ventral (V) retina, respectively, and perturbations of their expression lead to changes in expression of EphB receptors and their ephrin-B ligands that are critical for correct topographic mapping along this axis [4], [5]. A major decision for retinal axons along their path is whether to cross the midline or not. In binocular species, RGC axons extend to both the contra- and ipsilateral sides of the brain. The number of ipsilateral projecting RGCs and thus the area of the binocular region in the eye is different among species and depends on the visual field overlap between the two eyes. In mice, only about 3–5% of the total number of RGC project ipsilaterally, whereas the remaining 95–97% extend axons to the contralateral side [6]. This small ipsilateral RGC population originates from the periphery of the ventral-temporal retina, termed the ventral temporal crescent (VTC), and is marked by the expression of the transcription factor Zic2 and the receptor tyrosine kinase EphB1 [7], [8]. The choice for axons to segregate into crossed and uncrossed projections occurs at the ventral diencephalon. Ipsilaterally projecting RGC axons expressing EphB1 are repelled through interaction with its ligand ephrin-B2, which is expressed at the optic chiasm by the midline radial glia during the formation of ipsilateral projections [8], [9]. Within the region of the VTC in the retina, a precise genetic regulatory hierarchy exists to specify Zic2 positive and negative cells to control the magnitude of the binocular vision [10]. The exact molecular mechanisms however that regulate the generation of the two distinct retinal regions, a domain with a default to project ipsilaterally (within the VTC) and a domain with a default to project contralaterally (outside the VTC) are so far unclear. Similar to the retina, the region of the optic chiasm is a structure highly patterned by a number of factors, whose expression is crucial for its correct cellular organization and thus ensuring appropriate retinal axon trajectories [11].

MicroRNAs (miRNAs) are a class of small endogenous RNA molecules with important functions in gene regulation. These non-coding ∼21 nt long molecules bind to complementary sequences on protein-coding messenger RNAs and as a consequence direct their degradation and/or repress their translation [12], [13]. MiRNAs are evolutionary conserved and exhibit tissue-specific as well as developmental stage-specific expression [14], [15], suggesting important function in a variety of biological programs, including cell death, neuronal differentiation, synaptogenesis and other developmental processes [16], [17]. They are transcribed as long primary molecules mainly by RNA polymerase II [18]. These hairpin structures are then first processed in the nucleus by the RNase III-type protein Drosha [19] and its cofactor DGCR8 (DiGeorge syndrome critical region 8) [20], [21] resulting in pre-miRNAs which subsequently are exported to the cytoplasm by Exportin-5 [22]. The pre-miRNAs are then further processed by Dicer to generate mature miRNA molecules that are loaded into the RNA-induced silencing complex (RISC) ready to function as gene regulators [18]. Dicer is essential for embryonic development and in mice, null-mutant embryos die at embryonic day (E) 7.5 [23]. The generation of a conditional allele for *Dicer* made it possible to study its function in a tissue-specific manner [24].

Due to the broad extent of miRNAs functions during the development of the nervous system we speculated that they may be involved in the control of axon pathfinding decision at the midline. This could be either through indirect mechanisms, including regulation of factors important for tissue patterning, intracellular signaling or cell specification, or through direct regulation of axon guidance molecules critical for sensing the environment during axon extension. Indeed, functional RISCs have been found in developing axons [25] and studies in zebrafish deficient for miRNAs have indicated general axon pathfinding defects during development [26]. Although recent studies have looked at the roles of miRNAs in the eye, they did not analyze their contribution in processes important for establishing the correct connectivity during development [27], [28]. We therefore investigated the function of miRNAs for RGC axon outgrowth and pathfinding decisions in the visual system. In our approach we conditionally deleted *Dicer* in eye tissues using a Rx-cre mouse line [29] in conjunction with mice carrying a floxed conditional *Dicer* allele [24]. Our data show that early inactivation of *Dicer* in all cells of the developing visual system leads to pronounced RGC axon pathfinding defects at the optic chiasm. In addition, we find that the eyes are significantly reduced in size in homozygous *Dicer* mutant mice. However, both ventral retinal polarity and *Zic2* expression as a marker of the ipsilaterally projecting RGC population in the VTC remain unchanged. Our findings suggest an important function of miRNAs during axon extension towards the midline, directly or indirectly, to ensure the appropriate wiring of the nervous system during development.

## Results

### Conditional inactivation of *Dicer* leads to decrease of miRNA levels in retina and the ventral hypothalamus

**Figure 1:**
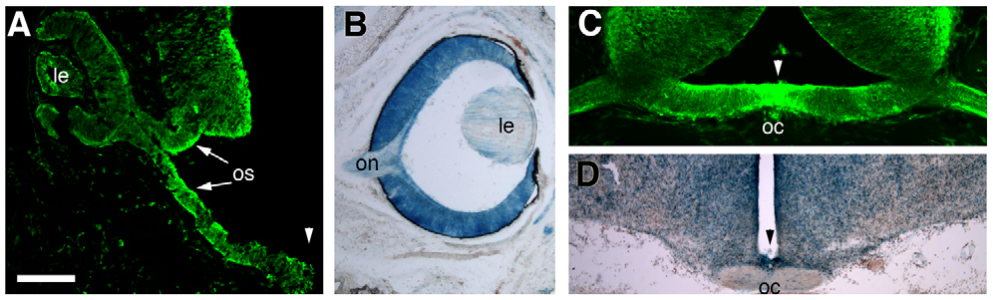
Cre-recombination occurs in the developing eye and the optic chiasm. Rx-cre transgenic mice were crossed to the R26R-EYFP and R26R-lacZ reporter lines to visualize regions in which cre is expressed. The EYFP (green) or ß-gal signal (blue) in the resulting offspring indicates Cre-mediated recombination. (A,B) Horizontal sections through E11.5 (A), and E17.5 (B) embryos. Strong cre expression is detected in all cells of the developing retina and optic stalk (arrows, os). (C,D) Coronal sections through the region of the optic chiasm (OC) at E13.5 (C) and E17.5 (D) shows strong reporter signal at the midline as well as the surrounding tissue. The arrowheads in (A, C, D) mark the midline. Le, lens; oc, optic chiasm; on, optic nerve; os, optic stalk. Scale bar = 100 µm in (A), 160 µm in (B,D), 200 µm in (C).

For our experiments we conditionally deleted Dicer in all cells of the developing retina and optic chiasm. To achieve this we used the Rx-cre transgenic mouse line [29], where the Cre recombinase is expressed under the control of an Rx promoter element. The Retinal homeobox gene Rx is an evolutionary conserved eye field transcription factor, essential for eye formation [30]. In mouse its expression starts at around E7.5 in the anterior neural plate and by E10.5 it becomes restricted to the retina and the ventral forebrain [30], [31]. To create mutant mice where *Dicer* is conditionally deleted in these tissues, we crossed the Rx-cre mouse line with a mouse line carrying a conditional null-allele for *Dicer* (*Dicer^fl/fl^*), where the exon containing most of the essential RNaseIII domain are flanked by *loxP* sites (floxed, fl) [24]. In these mice, Cre-mediated recombination results in a complete ablation of Dicer activity [24]. To confirm *cre* expression in the expected spatiotemporal pattern, we crossed the Rx-cre mouse line with the R26R-EYFP [32] and the R26R-lacZ [33] reporter lines in which Cre-mediated recombination leads to the removal of a stop-codon and subsequent EYFP and *lacZ* expression, respectively. As expected, we found EYFP/ß-gal positive cells at E11.5 in the developing visual system, including retina, optic stalks and at later stages at the forming optic chiasm, indicating Cre-mediated recombination in these areas (Figure 1 and Figure S1). In some cases we detected cre expression in the lens (Figure 1B), consistent with the original description of the Rx-cre transgenic mouse line [29]. Taken together, we concluded that the Rx-mouse line is suitable to conditionally delete *Dicer* in all the cells of the retina and the chiasm from the onset of their development.

**Figure 2:**
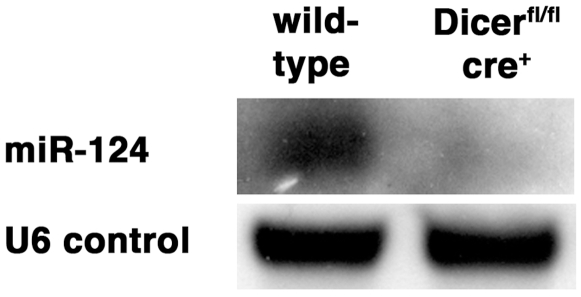
MiRNA levels are decreased in *Dicer* mutant mice. Northern blot analysis of mature miR-124 at E17.5 demonstrate a strong decrease in miRNA levels in Cre-positive tissues of homozygous *Dicer* mutant mice as compared to wild-type littermates. A probe against U6 RNA was used as a loading control and shows no difference between the genotypes. Nine micrograms of total RNA were separated, transferred to Nylon filters and probed with DIG-labeled LNA oligo probes.

Several reports have shown previously that the Cre-mediated recombination of the floxed *Dicer* allele leads to a loss of mature miRNAs [24], [27], [34]. To confirm that miRNAs levels were reduced in our case we performed Northern Blot analysis on isolated Cre-positive tissue from *Dicer* mutant and wild-type animals extracted at E17.5. Our results showed that Dicer inactivation resulted in a substantial loss of mature miRNAs as illustrated by the example of miR-124, the most abundant and broadly expressed miRNA in the nervous system [14] (Figure 2).

### Early deletion of Dicer leads to a severe size reduction of the eye

**Figure 3:**
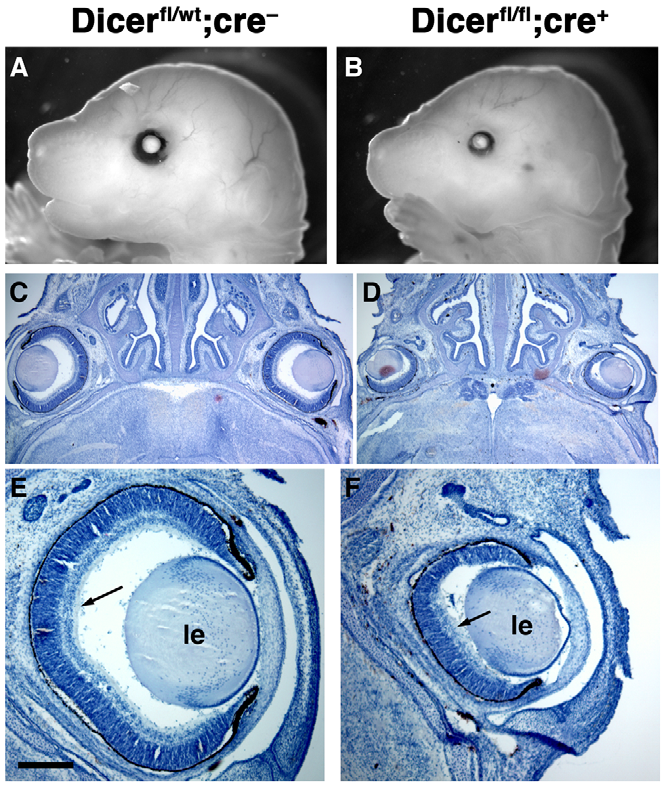
*Dicer* mutant animals show decreased eye size. (A, B) Lateral view of whole mount E17.5 embryos after tissues overlaying the eye have been removed to visualize size difference. (C, D) Horizontal sections through E17.5 heads and subsequent haematoxylin (blue) staining showing retinal layering and the difference in eye size. (E, F) Higher magnification of images in (C,D). Arrows indicate RGC layers. le, lens. Scale bar = 2.5 mm in (A,B); 1 mm in (C,D); 300 µm in (E,F).

**Figure 4:**
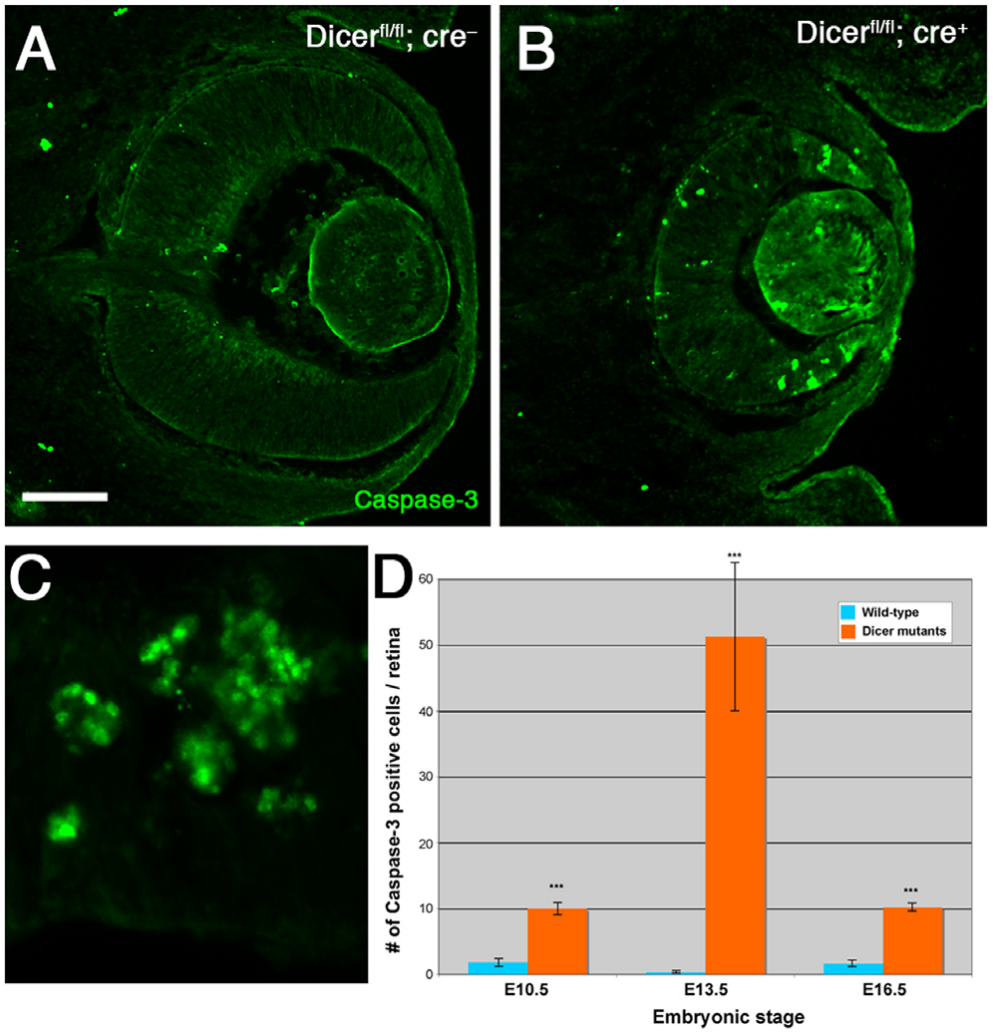
Dicer deletion leads to increased cell death in the retina. Immunohistochemical staining for caspase-3 (green signal) on horizontal sections through the eye of E13.5 wild-type (A) and *Dicer* mutant mice (B, C). Increased number of caspase-positive cells were found in the retina of Dicer deleted mice. (C) Higher magnification of a retinal region at E13.5 containing several apoptotic cell rosettes. (D) Quantification of caspase-3 positive cell counts in *Dicer^fl/fl^; cre^–^* and *Dicer^fl/fl^; cre^+^* mice (for both genotypes n = 7 at E10.5, n = 6 at E13.5 and E16.5, respectively, where n is the number of retinae analyzed). Error bars represent SEM. Values are statistically significant using the Students't-test (p<0.007) for all three ages. Scale bar = 100 µm in (A,B); 20 µm in C.

In mouse, eye development starts with the specification of the eye field in the anterior neural plate at E7.5 and the formation of the eyecup is completed with the closure of the ventral fissure at around E12.5 [35]. We found that the homozygous *Dicer* mutant mice were clearly distinguishable from wild-type or heterozygous littermates from around E13 onwards due to an alteration of eye size. This phenotype was 100% penetrant (n>35). The eyes laid much deeper in the head than seen normally and, when the overlaying eyelid tissue was removed, a substantial reduction in overall eye size became apparent in homozygous Dicer^fl/fl^; cre^+^ embryos (Figure 3). In heterozygous littermates we found that eye size and positioning was normal (not shown). Coronal sections through the eye at E17.5 indicated that in Dicer-deficient mutant mice the vitreous humor was considerably reduced and the lens was not placed inside the retinal cup enclosed by the ciliary margin (CM), but it rather stuck out of the cup with the CM about half way at the ‘equator’ of the lens (Figure 3F). At earlier embryonic stages we found that the lens had only somewhat displaced towards the outside of the eye compared to wild-type (for example as shown for E13.5 in Figure 4A/B), suggesting that this phenotype may be due to a secondary effect based on the increasing disproportion in size of the retinal cup and the lens that causes its protrusion in later stages. Despite the size differences, the gross morphology of the mutant retina looked very similar to wild-type (Figure 3F).

### Deletion of Dicer increases apoptosis in the retina

The reduction in overall size of the retina in *Dicer* mutant mice could be due to induced cell death in this tissue. Indeed, several recent reports have shown that conditional deletion of *Dicer* leads to an increase in apoptotic cells in the affected structures [34], [36]. We therefore performed immunohistochemical analysis using an antibody raised against the cleaved form of Caspase-3, which is a marker for cells undergoing programmed cell death, on retinal sections. Manual counts of the stained cells showed a significantly higher proportion of Caspase-3 positive cells in the *Dicer* mutant mice, compared to wild-type or *Dicer^fl/wt^; cre^+^* mice (Figure 4). Quantification indicated that in *Dicer^fl/fl^; cre^+^* mice the increase of apoptotic cells is around 5-fold at E10.5 (n = 7 retinae for each genotype) and E16.5 (n = 6) and around 50-fold, at E13.5 (n = 6), compared to Cre-negative littermates (Figure 4D). The higher difference at E13.5 is most likely due to the fact that at this stage we detected a large number of Caspase-3 positive cell-rosettes (Figure 4C). Such rosettes are indicative of retinal cell death and have been found in both mouse and *Xenopus* retinae after deletion of Dicer [27], [28]. The differences we observed between mutant and wild-type littermates were statistically significant, with p-values <0.007 (Students't-test). These data suggest that miRNAs are essential for the survival of retinal cells and Dicer deletion leads to increased apoptosis responsible -at least in part- for the reduction in overall size of the eye.

### The formation of the optic disc and correct RGC axon exit from the eye is independent of Dicer

**Figure 5:**
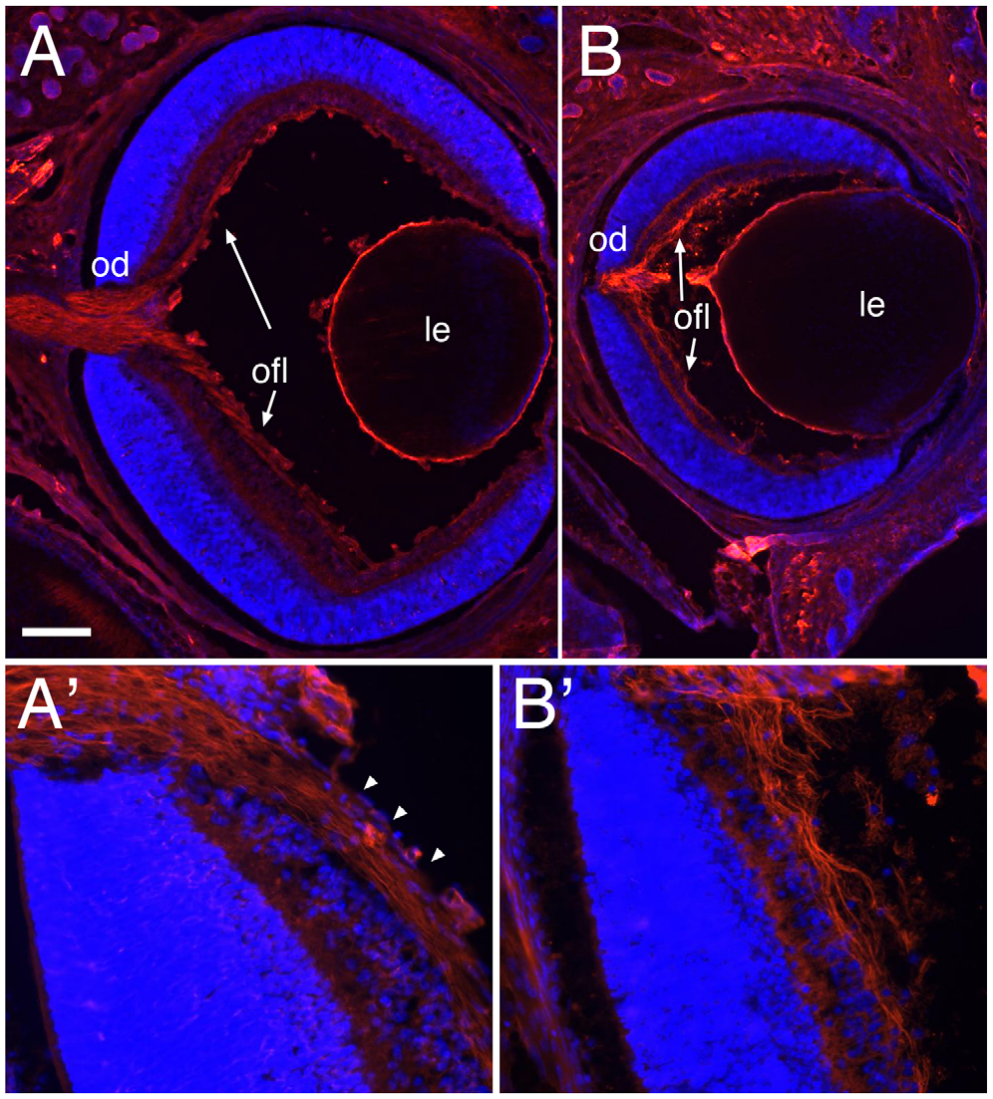
RGC axons in the optic fiber layer. Coronal sections through eyes of P0 wild-type (A, A′) and homozygous Dicer mutant mice (B,B′), followed by immunohistochemistry using a neurofilament antibody (red signal) and counterstaining with Hoechst (blue). (A, B) RGC axons are extending in the optic fiber layer (ofl, arrows) and exit the eye through the optic disc (od) in both wild-type (A) and *Dicer* mutant mice (B). (A′, B′) Higher magnifications of images shown in (A, B) reveal fasciculated and straight axon extensions in wild-type retina (A′, arrowheads), whereas axons show defasciculation and aberrant growth in *Dicer* mutant mice (B′). Le, lens; od, optic disc, ofl, optic fiber layer. Scale bar = 200 µm (A, B); 50 µm (A′, B′).

Further investigation of the eye morphology in Dicer^−/−^ mice showed no defects in the formation of the optic disc, the exit point for RGC axons from the retina. In 100% of the mutant mice the optic disc was closed and properly formed (Figure 5A, B).

Previously it had been shown that the interaction of Slit1 and Slit2 via their receptor Robo2 is critical for correct intraretinal axon guidance and in mice carrying mutations in these genes, RGC axons are no more correctly extending into the optic fiber layer [37], [38]. We therefore investigated if the deletion of miRNAs leads to a similar phenotype. Immunohistochemical analysis of retinal sections using antibodies against neurofilaments showed that in Dicer mutant mice RGC axons extend correctly along the optic fiber layer, without growing aberrantly into other retinal layers (Figure 5). However, we detected aberrant growth morphology of RGC axons in the optic fiber layer. The axons appeared defasciculated and ‘wavy’ while growing towards the optic disc (Figure 5B′), in contrast to the straight and fasciculated appearance of axons in the optic fiber layer of wild-type mice (Figure 5A′).

### Dicer deficient mice exhibit strong axon pathfinding defects at the midline

**Figure 6:**
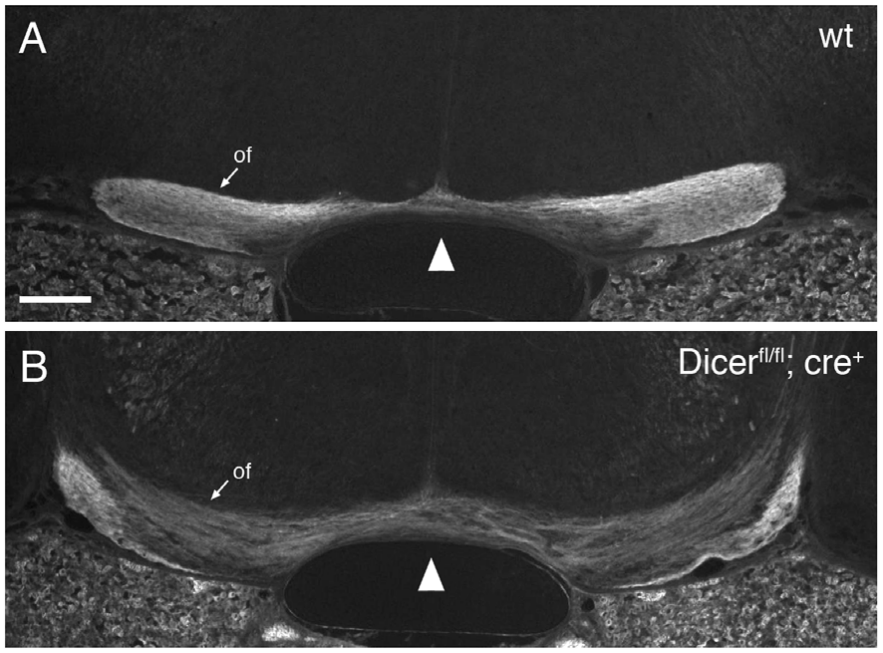
The optic chiasm is disorganized in *Dicer* mutant mice. Horizontal sections through the optic chiasm of E17.5 wild-type (A) and *Dicer* mutant (B) embryos, followed by immunohistochemistry using Neurofilament antibody. The midline is marked by the arrowhead in both panels. (A) In wild-type embryos the optic fiber bundles (of) are crossing at the chiasm and extend further around the ventral diencephalon. (B) In *Dicer* mutant embryos, axons are defasciculated and extend not only along the periphery of the diencephalon, but appear also deeper (more dorsal) in the tissue. Of, optic fibers. Scale bar = 200 µm.

**Figure 7:**
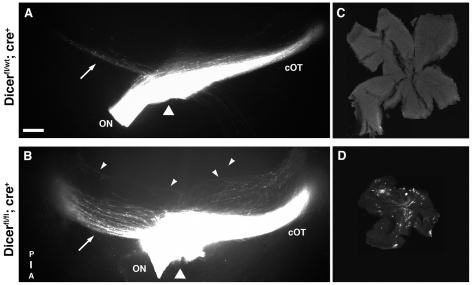
Dicer loss leads to severe axon pathfinding defects at the optic chiasm. (A, B) Ventral view of the optic chiasm under fluorescent microscope after labeling all RGC axons from one eye with the lipophilic dye DiI in E18.5 heterozygous (A) and homozygous (B) *Dicer* mutant mice. The triangles depict the midline, anterior is down and posterior is up. (C, D) Corresponding contralateral flat-mounted retina from heterozygous (C) and homozygous (D) *Dicer* mutant mice. (A) In heterozygous *Dicer^fl/wt^; cre^+^* mutants the majority of axons crosses to the contralateral side, whereas only a small proportion of axons stay ipsilaterally (arrow), as in wild-type mice. The fibers form a fasciculated contralateral optic tract (cOT). (B) In homozygous *Dicer* mutant mice the proportion of axons projecting to the ipsilateral side is significantly increased (arrow). In addition, a large number of fibers can be found extending outside the region of the optic chiasm (arrow heads) toward the posterior part of the brain. They then turn either ipsi- or contralaterally to extend further dorsally over the diencephalon, however they do not fasciculate with the main optic tract (cOT). (C, D) The corresponding retina contralateral to the DiI placement show back-labeled cells and extended axon fibers only in the *Dicer* null mice (D), but not in the heterozygous mutants (C). ON: optic nerve; OT: optic tract. Scale bar = 150 µm in (A,B); 200 µm in (C,D).

The aberrant morphology of axons in the optic fiber layer suggested a role of miRNAs in axon extension. We therefore investigated next if the RGC axons that exit the eye show any aberrant projection pattern along the visual pathway. One of the major choice points for RGC axons on their journey to their targets is the midline at the ventral hypothalamus, where in mice the majority of axons cross to the contralateral side and only 3–5% of the axons stay ipsilaterally [6]. Most of these decisions take place between E13 and E17.5 during development [11]. We wanted to know if miRNAs play a role in this process and therefore concentrated on the RGC projection pattern at the optic chiasm. For this we first performed immunohistochemical neurofilament staining on coronal sections through the region of the chiasm of P0 *Dicer* mutant and wild-type littermates. Our analysis showed that in contrast to the wild-type situation where the RGC axons are highly fasciculated across the chiasm and in the optic tract, the *Dicer* mutant mice were missing such dense axon bundles (Figure 6). Instead, the axons seemed defasciculated and dispersed into the ventral hypothalamus (Figure 6B). Immunohistochemical staining of RGC axons using antibodies against the cell adhesion molecule L1 showed a similar phenotype at the chiasm (Figure S2). Interestingly, the overall staining intensity was much lower in homozygous Dicer mutants, suggesting a possible downregulation of L1 in retinal axons. These results indicated that the lack of miRNAs disturbed the organization of the RGC axon trajectories at the midline. To investigate this finding in greater detail we labeled retinal axons from one eye of E18.5 wild-type and *Dicer* mutant littermates with the lipophilic dye DiI (Figure 7). At E18.5, most axons originating in the retina have already passed the chiasm and have made the decision to project either ipsi- or contralaterally to form the optic tract on either side of the brain [39]. We found that in wild-type mice the axons extended along their normal pathway toward the midline where the majority crossed to the contralateral side (Figure 7A) and only a small proportion of axons entered the ipsilateral optic tract to reach targets on the same side of the brain. In contrast, our analysis of the *Dicer^fl/fl^; cre^+^* mutant mice showed that, although the axons extended normally toward the midline, a number of severe abnormalities were apparent at the optic chiasm (Figure 7B). One of the most striking phenotypes was the drastically increased number of fibers that failed to cross the chiasm and projected ipsilaterally (arrow in Figure 7B). These axons extended further towards the midline before turning ipsilaterally, resulting in a broader distribution of fibers along the entire width of the ipsilateral optic path (compare DiI positive axons indicated by arrow in Figure 7A and B). Moreover, we found many axons that overshot the chiasm and extended further posteriorly along the midline (arrowheads in Figure 7B). Some of them then turned laterally toward the ipsilateral or contralateral side, however this population of overshooting fibers did not fasciculate with the normal optic tract generated by the rest of the axons, but formed a secondary optic tract. This was particularly evident on the contralateral side (Figure 7B). We also detected aberrant branching for some of the axons leaving the chiasm. Since in Dicer mutants overall less axons were present, we raised the gain of the image depicted in Figure 7B to be able to visualize the extension of single axon aberrancies. Finally, in the homozygous conditional *Dicer* mutant mice a substantial proportion of fibers extended into the contralateral optic nerve, after they had crossed the midline. Such pathfinding errors can be seen occasionally in wild-type mice, however only for a few RGC axons. To analyze how far these ectopically projecting axons extended we examined the contralateral retina for any presence of dye labeling (Figures 7C and D). Indeed, we found a substantial amount of DiI-positive regions in *Dicer^fl/fl^; cre^+^* mice, indicating that fibers are extending far into the contralateral retina. DiI is distributed anterogradely as well as retrogradely, thus both terminating fibers (anterogradely labeled from the experimental eye) and cell bodies (retrogradely labeled from the experimental eye) were detected. In contrast, in wild-type and heterozygous *Dicer^fl/wt^; cre^+^* littermates we did not detected any labeled fibers extending into the contralateral retina (Figure 7C).

**Figure 8:**
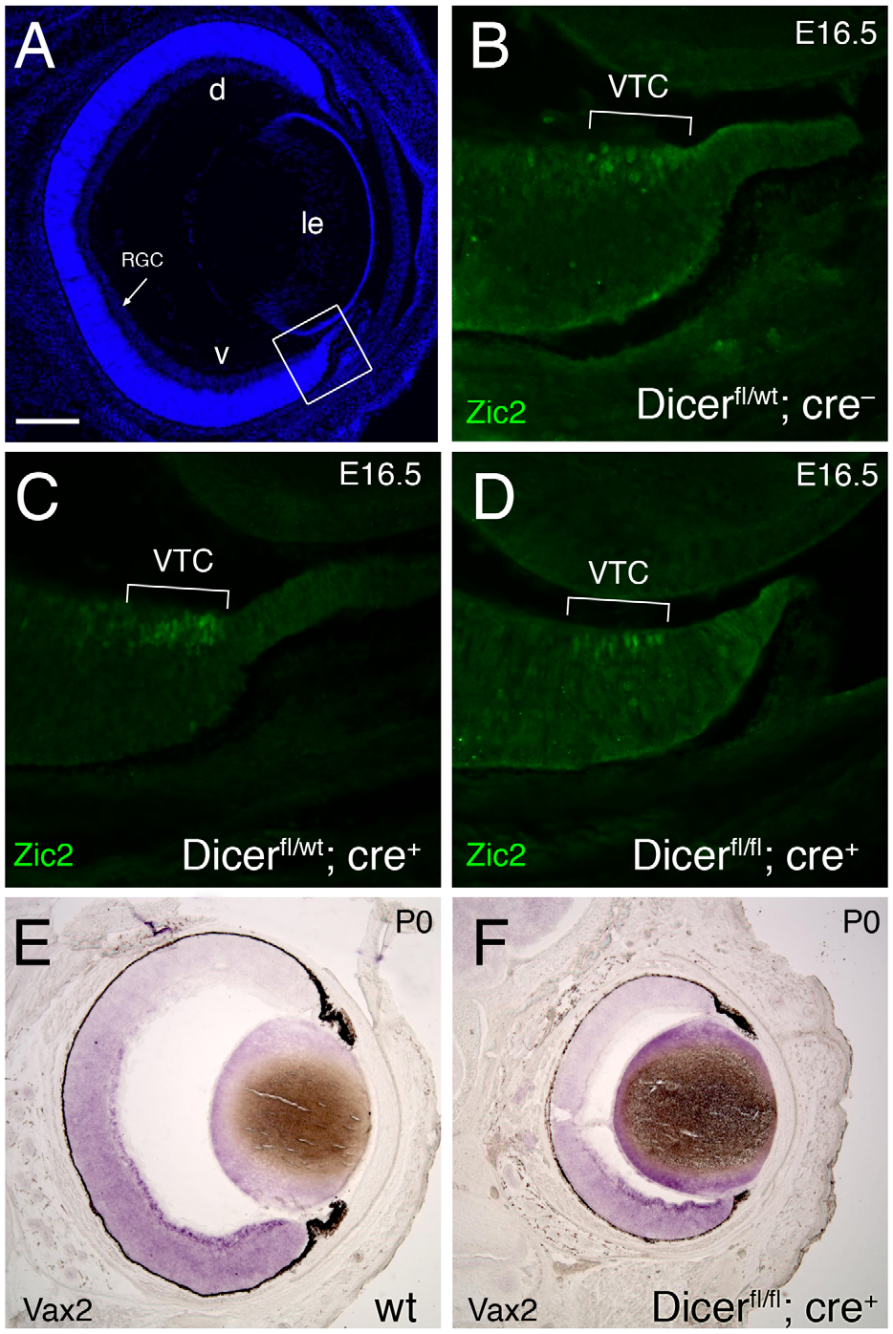
Zic2 and Vax2 expression are unchanged in Dicer mutant mice. Coronal sections through retinae at E17.5 (A–D) after immunohistochemistry against Zic2 and P0 (E,F) after in situ hybridization with an antisense probe against *Vax2*. (A) Overview of a section stained with Hoechst (blue signal). Dorsal (d) is up and ventral (v) is down for all panels showing the entire retina. The boxed area, shown in higher magnification in (B–D), is the location of the ventral temporal crescent (VTC) in the retina. (B–D) Zic2-positive cells are restricted to the VTC in wild-type (B), hetero- (C) and homozygous (D) Dicer mutant mice. (E,F) The ventral marker *Vax2* is present in strong gradients from high ventral to low dorsal in both wild-type (E) and *Dicer^fl/fl^;cre^+^* mice. d, dorsal; le, lens; RGC, retinal ganglion cells; v, ventral; VTC, ventral temporal crescent. Scale bar = 200 µm (A); 50 µm (B–D); 300 µm (E,F).

In binocular species, the proportion of ipsilateral projecting RGC axons is consistent with the number of *Zic2* expressing cells and the area of their origin in the retina [7]. In mice the Zic2-positive RGCs are restricted to the VTC, which is the only region of visual overlap with the other eye [6]. Furthermore, it has been shown that misexpression of this transcription factor in the retina is sufficient to change the trajectory of axons from crossed to uncrossed [40]. This raised the possibility that the marked increase of ipsilaterally projecting RGC axons we detected in Dicer mutant mice was due to a general upregulation of Zic2 in the retina. We therefore analyzed Zic2 expression from wild-type and Dicer mutant mice at E16.5 (Figure 8A–D) using a Zic2 specific antibody [41]. In coronal sections through the retina we found that Zic2 expression was unchanged in both hetero- (Figure 8C) and homozygous (Figure 8D) Dicer mutants, as compared to wild-type mice (Figure 8B) and still was restricted to the VTC only. We did not detect any Zic2-positive cells outside the VTC region. In addition, to investigate overall ventral polarity of the retina we used labeled riboprobes specific against *Vax2*, a ventrally expressed homeodomain transcription factor [3]. In situ hybridization experiments on coronal retinal sections showed a strong high V to low D gradient of *Vax2* expression in Dicer mutant mice, confirming that the overall ventral polarity is still defined (Figure 8E, F).

Taken together, our results demonstrate that the lack of miRNAs through the removal of Dicer leads to a series of severe RGC axon pathfinding defects at the optic chiasm independent of changing ventral polarity of the retina or the origin and number of Zic2-positive cells in the VTC.

## Discussion

**Figure 9:**
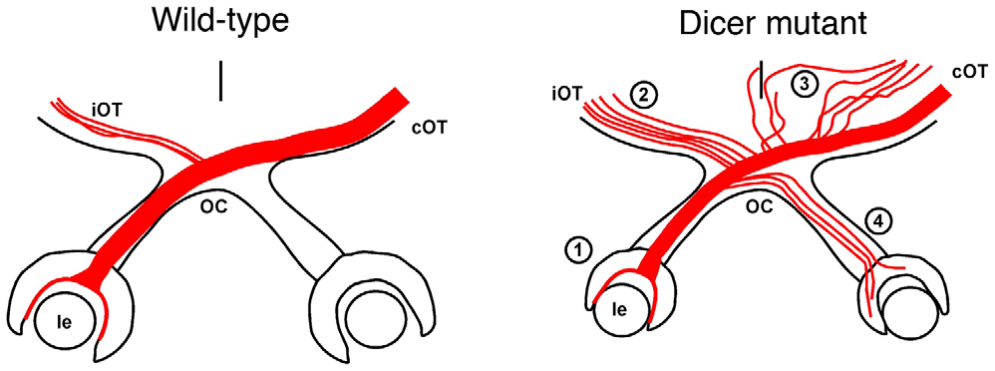
Diagram summarizing the observed phenotypes in conditionally deleted Dicer mice. In wild-type or heterozygous *Dicer* mutant mice (left diagram), the neural retina encapsulates the lens (le) and RGC axons (red) extend toward the midline, forming the optic nerve. At the chiasm (OC) most of the axons cross to the contralateral side (cOT), whereas only 3–5% stay ipsilaterally (iOT). The axons form tight fasciculated axon bundles at the point of crossing and when extending into the optic tracts. In homozygous *Dicer* mutant (right diagram) we detect four major defects: (1) the eye is smaller, with the lens being only about 50% encapsulated by the retina; (2) an increased ipsilateral projection; (3) fibers extend further than the optic chiasm and then, after turning, they form a parallel optic tract (4) a significant proportion of axons extend aberrantly into the contralateral eye.

The ability to process visual information appropriately is dependent on the correct establishment of the necessary circuitry that connects the retina with its targets in the brain. In this study, we provide evidence that the miRNA family of small non-coding RNA molecules are important regulators of eye development and axon pathfinding decision and therefore are critical for the correct establishment of the visual pathways. By crossing a mouse line containing floxed alleles for *Dicer*, a critical protein for the generation of mature miRNAs, with a Rx-cre transgenic mouse line, we conditionally deleted Dicer in all the cells of the retina and ventral hypothalamus from the initiation of their development. We show that homozygous *Dicer* mutant mice exhibit severe axon guidance defects in the visual system, including an increased ipsilateral projection, axons extending ectopically outside the region of the optic chiasm, the formation of a secondary optic tract, and aberrant growth of RGC axons into the contralateral optic nerve and eye. In addition, the mutant mice show a microphthalmia phenotype. A summary of all the phenotypes we detected is shown in Figure 9.

### Early eye formation is independent of miRNAs

The expression of the eye field transcription factor *Rx* starts at around E7.5 in the anterior neural plate [30]. It has been shown that Rx is essential for the formation of retinal progenitor cells [42] and mice containing null mutations of the *Rx* gene do not develop any morphologically visible eye structures [30]. These results demonstrated that *Rx* is expressed and required from the initial stages of eye development. For our experiments we used transgenic mice in which the Cre-recombinase is expressed under the control of the Rx-promoter element [29], thus we generated a conditional *Dicer* deletion starting at the initiation of early eye development, days before the optic cups are even formed [35]. However, in 100% of the homozygous mutant embryos we found that the eyes are able to form completely, albeit smaller. The correct patterning of the optic fissure is dependent on the actions of Bmp7 and Shh [43] and mutations in *Pax2* [44] and *Vax1* [45] lead to a deficiency in the closure of the optic fissure, a condition known as coloboma. We did not find any defects in the closure of the optic disc in conditional *Dicer* mutant mice. Overall, these findings suggest that the early morphogenic processes of eye development, including the evagination of the diencephalon, the formation of the optic vesicles and the optic cups, the formation of the lens, and closing of the optic fissure, are independent of Dicer function and regulation through miRNAs. However, we detected in *Dicer^fl/fl^; cre^+^* mice an increase in apoptosis in the retina. These results are consistent with other reports where the conditional deletion of Dicer for example in the cortex [34], limb bud [24] or lung epithelium [46] resulted in a similar increase of apoptotic cells and subsequently in a reduction in cell number and tissue size. Therefore, this suggests that programmed cell death might be, at least partially, responsible for the observed microphthalmia phenotype. The timing of miRNA depletion is important though: It has been shown that Dicer removal early in development leads to a more pronounced increase in apoptosis, whereas later removal has no effect [34], [47]. The substantial reduction of eye size we detect is thus consistent with these findings, since we are deleting Dicer from the beginning of eye development.

A recent report describes a knockdown of *Dicer* in *Xenopus* retinal progenitor cells using morpholino oligonucleotides [28]. In addition to an increased number of apoptotic cells and the formation of rosette-like structures, the authors find also a change in cell-cycle control during retinogenesis. As a result, the eyes of injected animals were smaller and also exhibited strong lamination defects [28]. In mice, where *Dicer* has been conditionally deleted using the Chx10Cre transgenic line (“CKO” mice), the initial retinal lamination during development was normal [27] but the authors detected retinal degeneration phenotypes, including the formation of photoreceptor rosettes, at more mature ages starting at P16 [27]. In contrast, we detected apoptotic cells arranged in rosettes in retinae of Dicer mutant mice already at E13.5. A possible explanation for this difference in the onset of degeneration might also be the extent of Dicer deletion in the eye (besides the timing issue of cre-expression, discussed above). In the CKO mouse line the Cre-recombinase is expressed in a mosaic fashion in the eye [48] leading to an incomplete deletion of Dicer in the retina with most of the RGCs for example still Dicer-positive [27]. In contrast, the Rx-cre line used in this study eliminates Dicer in all retinal cells, including the RGCs and as a consequence possibly leads to more pronounced degeneration.

### Dicer deletion leads to axon pathfinding defects at the midline

The perturbation of the appropriate formation of axon tracts upon Dicer loss has been reported during cortical development in mice and in trigeminal neurons in zebrafish [26], [34], [36], [49]. Indeed, since the Rx promoter drives cre-expression also in the cortex (Figure S1), we noticed that homozygous *Dicer^fl/fl^;Rx-cre^+^* mutants are missing major forebrain commissures (data not shown) and we are currently analyzing this in greater detail. In addition, functional RNA-induced silencing complexes have been found in axons and growth cones in culture [25], supporting the hypothesis that miRNAs are involved in axon guidance decisions. We show here that early and complete deletion of Dicer in retina and ventral hypothalamus leads to severe pathfinding defects of RGC axons at the optic chiasm. Many of the axons become misrouted at the midline and exhibit a combination of varying aberrant projection patterns. It has been shown that miRNAs not only repress, but also can upregulate translation [50], as well as altering the transcriptome [51], leading in combination to large changes in protein output of a cell [52], [53]. Therefore the detected phenotypes could be based on both these types of changes affecting directly or indirectly the control of pathfinding, patterning and other mechanisms. Secondly, because we used a mouse cre-line that conditionally deletes *Dicer* in both, the retina and at the optic chiasm, the defects we detect could be based on miRNA action in either of these tissues or both. However, it is interesting to note that the extensive axon pathfinding errors we observed partially phenocopy some defects that have been previously described in studies where individual genes expressed in these regions were deleted.

Increased ipsilateral projections have been found in a number of reports examining effectors at the optic chiasm. This structure is precisely patterned, creating longitudinal molecular subdivisions of the forebrain in which RGC axons run parallel to cross from one side to the other [54]. These subdomains are crucial to channel axons by repulsive and attractive mechanisms in order to ensure their correct guidance through the chiasm [11]. In zebrafish, a monocular species with 100% of the RGC axons projecting to the contralateral tectum, mutations in the lim-homeodomain transcription factor lhx2 (*belladonna* mutation) cause mispatterning of the forebrain and expression changes in regulatory genes as well as axon guidance molecules, such as *sema3d*, *netrin1a* and *slit2* [55]. As a consequence, RGC axons fail to cross the midline and project ipsilaterally only. In mouse, the loss of the winged helix transcription factors Foxd1 and Foxg1, both expressed in non-overlapping regions at the chiasm, lead to an aberrantly larger proportion of ipsilateral projecting RGC axons [56], [57], again partially due to a mispatterning of the chiasmatic region. However, both of these transcription factors are also prominently expressed in the eye (Foxd1 in VT retina, Foxg1 in nasal retina) and the studies suggest, at least in part, a RGC-autonomous effect [56], [57]. Furthermore, it has been shown that changes of sonic hedgehog activity in vivo at the chiasm alter the decussation of RGC axons [58], [59]. Whether the deletion of Dicer leads to any changes in patterning of the ventral diencephalon and thereby causing the RGC axons to misproject, has yet to be established.

The retina is patterned along both its axes, in addition to be subdivided into a binocular (the VTC) and monocular (the remainder of the retina) region. This is achieved through the combinatorial action of morphogens and transcription factors that subsequently control the expression of axon guidance molecules in RGCs [1]. Several of those genes have been shown to influence axon divergence at the chiasm. For example deletion of Isl2 [10] or NrCAM [60], as well as overexpression of Zic2 [40], EphB1 [61] and Boc [62] lead to an increase in ipsilateral projections. Interestingly, we find that the deletion of *Dicer* in the eye has no effect on the overall ventral identity of the retina or on the expression of Zic2 in the VTC. This suggests that the specification of ipsilaterally projecting RGCs and the patterning along the DV axis occurs independent of miRNAs. Additional marker analysis is needed to confirm if this holds true also for the nasotemporal axis of the eye.

Aberrant axon growth into the contralateral optic nerve similar to what we detected in our *Dicer* mutant mice, has been reported in *Slit1/Slit2* double mutant mice [63] and in mutants affecting the heparan sulfate proteoglycan sulfation pathway [64], [65]. *Slit1* and *2* are both expressed at the region of the chiasm and create a non-permissive boundary for RGC axons, therefore channeling the fibers correctly along their path across the midline [66]. The lack of these boundaries in *Slit1/2* double mutant mice allows axons to extend aberrantly outside their normal trajectories [63]. The extension of RGC axons into the diencephalon and the contralateral optic nerve resembles very closely our findings in the *Dicer* mutant mice. Similarly, the mutation of the Slit receptor, Robo, leads also to aberrant projection patterns at the ventral hypothalamus [67], [68]. However, the phenotypical overlap between the *Dicer* mutants and the *Slit/Robo* mutants is not 100%, since we do not observe any premature midline crossing or any intraretinal guidance defects of RGC axons [37], [38], [63].

The similarities between our results and the phenotypes caused by these individual gene alterations described above imply that miRNAs control the same or at least related pathways influencing RGC axon guidance decisions at the midline. The extent however and the range of phenotypes that we detect in the *Dicer^fl/fl^; cre^+^* mutant mice, suggest that the expression of multiple genes in retina, chiasm or both has been perturbed.

Several studies have investigated the expression patterns of miRNAs in the retina and identified a substantial number of retina-specific members of these non-coding RNA molecules [69], [70], [71], [72], [73], [74]. Different bioinformatics algorithms predict a high number of potential target genes for these miRNAs, including some of the genes known to be important for axon guidance at the midline described above. However, at present it is unclear which miRNAs and their target genes are most important for retinal development and/or axon guidance function. A recent report describes the targeted deletion of miR-182, a highly abundant miRNA in the retina [75]. Yet, the mutant mice did not show any obvious retinal defect. These results suggest that similar to the combinatory action of transcription factors, a combination of different miRNAs may be responsible for the correct regulatory mechanisms needed during visual system development.

Our work demonstrates in vivo the important role of miRNAs for the correct navigation of RGC axons through the optic chiasm. Although the exact mechanism how these short RNA molecules affect axon pathfinding is not yet known, we show here that they are critical for the establishment of the circuitry during the development of the nervous system. Furthermore, the defasciculation of RGC axons and their aberrant appearance within the retina suggest, at least in part, a cell-autonomous function for miRNAs during axon extension and pathfinding. Now it will be important to extend investigations in order to further identify not only the particular miRNAs involved in this complex regulatory network, but also the target genes that are affected.

## Materials and Methods

### Ethics statement

All animal procedures were carried out according to institutional guidelines and protocols approved by the KCL Ethics Committee and the Home Office.

### Animals

*Dicer^fl/fl^* mice [76] and the R26R-*lacZ* reporter line [33] were purchased from Jackson Laboratory, the Rx-cre transgenic mouse line [29] was kindly provided by Milan Jamrich and the R26R-EYFP reporter line [32] was kindly provided by Frank Costantini. Genotyping of floxed *Dicer* mice was performed by PCR as described [24]. To genotype the Rx-Cre mouse line, we used PCR primers MeRxgenoF (GTTGGGAGAATGCTCCGTAA) and MeRxgenoR (GTATCCCACAATTCCTTGCG) to amplify a 362 bp product detecting presence of cre sequences. DNA was isolated from the tail tissue of each mouse embryo, using 50 mM NaOH for 30 minutes at 95°C followed by 1 M Tris buffer, pH 8 at room temperature. 1 µl samples of DNA were used for PCR genotyping. Amplification conditions were: 2 min at 94°C, 94°C 30 sec, followed by 32 cycles of 94°C for 30 sec, 60°C for 1 min, then 72°C for 1 min, followed by a final 72°C extension for 7 min.

### Immunohistochemistry and tissue staining

Staged mouse embryos were dissected and fixed in 4% paraformaldehyde/PBS overnight at 4°C. After infiltration with 20% sucrose the samples were embedded and frozen in OCT medium (Tissue-Tek). For histochemistry and immunolabeling, 16–18 µm tissue sections were cut on a cryostat and processed according to standard protocols. The working dilutions and sources of antibodies used in this study were: rabbit anti Caspase-3 antibody (1∶300, Abcam), rat anti-L1 monoclonal antibody (1∶100, Millipore/Chemicon), mouse anti-Neurofilament 2H3 (1∶10, Developmental Studies Hybridoma Bank), rabbit anti Zic2 (1∶10'000, [41]). Alexa-conjugated secondary antibodies (Alexa Fluor-488 and -568, Invitrogen) were used at a concentration of 1∶400. Cell nuclei were counterstained with Hoechst (bis-benzimide) in 1∶1000 dilution (Molecular Probes). Sections were then analyzed under fluorescent illumination. Beta-galactosidase expression was detected using histochemical x-gal staining. 20 µm thick sections from E17.5 mice heads were fixed in 2% paraformaldehyde in 1.0 M PBS (pH 7.4) at 4°C for 10 min. Sections were washed in PBS-2 mM MgCl2 twice for 10 min at 4°C and followed by detergent wash (2 mM MgCl2, 0,01% Na-deoxycholate and 0.02% NP-40 in 0.1 M PBS pH 7.3) twice for further 10 min at 4°C. Slides were then incubated in freshly prepared x-gal staining solution (1.33 mg/ml x-gal in pre-warmed detergent wash solution 5 mM potassium ferricyanide, and 5 mM potassium ferrocyanide) at 37°C in the dark in humidified chamber for approx. 1 hour or until staining was sufficiently detected. Slides were then washed in PBS 2 mM MgCl2, and coverslipped with Fluoromount G.

### Axonal tracing using lipophilic dye

Embryos were fixed with 4% PFA in PBS and crystals of DiI were placed unilaterally at the optic nerve head. Heads were incubated at 30°C in 4% PFA in PBS for 7–14 days. Brains with the optic nerves intact were carefully removed from the head and the region of the optic chiasm was analyzed en face under fluorescent illumination. The eye, contralateral to the DiI crystal placement was removed and the retina was flat-mounted, RGC layer up and analyzed for dye signals under fluorescent illumination.

### Imaging

All brightfield and fluorescent imaging was done on a Zeiss Axioskop with a cooled monochrome CCD camera (Retiga EXi Blue) and a Leica Stereomicroscope M165FC with a cooled color CCD camera (QICAM) in combination with Volocity Acquisition software (Perkin Elmer).

### Northern blot analysis

Total RNA was purified from tissue using TRIZOL (Invitrogen) according to manufacturers instructions and 8 µg for each genotype were subsequently resolved by 15% TBE-Urea polyacrylamide gel electrophoresis (PAGE). Gels were stained with Ethidium Bromide and photographed under UV illumination. RNA was transferred by electroblotting onto Nylon + membranes (Invitrogen) and then crosslinked by UV (Stratalinker) and baking at 80°C for 30 minutes. Filters were pre-hybridised at 45°C for 30 minutes in Ultrahyb-Oligo-Hybridisation buffer (Ambion) before ready DIG-labeled miRCURY LNA probes (Exiqon, 10 pmol) were added and hybridized overnight. Filters were then washed at 45°C 2×10 min with 2xSSC, 0.1% SDS and 1×10 min with 1xSSC, 0.1%SDS. Subsequent washes and chemiluminescent DIG signal detection was carried out using the DIG detection starter Kit (Roche) according to manufacturers instructions. Blots were stripped by incubation in 1%SDS at 85°C for 30 min for reprobing.

## Supporting Information

Figure S1 Cre-expression pattern in Rx-cre mice. Serial coronal sections through E17.5 heads of Rx-cre; R26R-lacZ mice after x-gal staining. The blue signal indicates expression of cre. (A) Schematic showing the level of sections along the anterior-posterior axis depicted in the other panels. (B–F) Cre expression is strongest in the retina (B,C) but also visible in more broadly in structures within the forebrain.
(4.49 MB TIF)

Figure S2 Disorganization of retinal fibers at the midline in *Dicer* mutant mice. Horizontal sections through the optic chiasm of E17.5 wild-type (A–E) and *Dicer* mutant (F–J) embryos, followed by immunohistochemistry using L1 antibody (brown staining) and counterstaining with haematoxylin (blue). (A–E) In wild-type embryos the retinal fiber bundles are crossing at the chiasm and extend further around the ventral diencephalon (arrows), leading to strong L1 staining. (F–J) In *Dicer* mutant embryos, L1 staining is much weaker across the entire chiasm, possibly because axons have defasciculated. L1-positive fibres can be found not only at the surface of the diencephalon, but also deeper in the tissue (arrowheads). v, ventral; d, dorsal. Scale bar = 200 µm.
(10.02 MB TIF)
