# Supplementary figures and images for: Perturbations of MicroRNA Function in Mouse Dicer Mutants Produce Retinal Defects and Lead to Aberrant Axon Pathfinding at the Optic Chiasm
Source: PLoS One. 2010 Apr 1;5(4):e10021. doi: 10.1371/journal.pone.0010021 (PMC2850387; doi:10.1371/journal.pone.0010021)

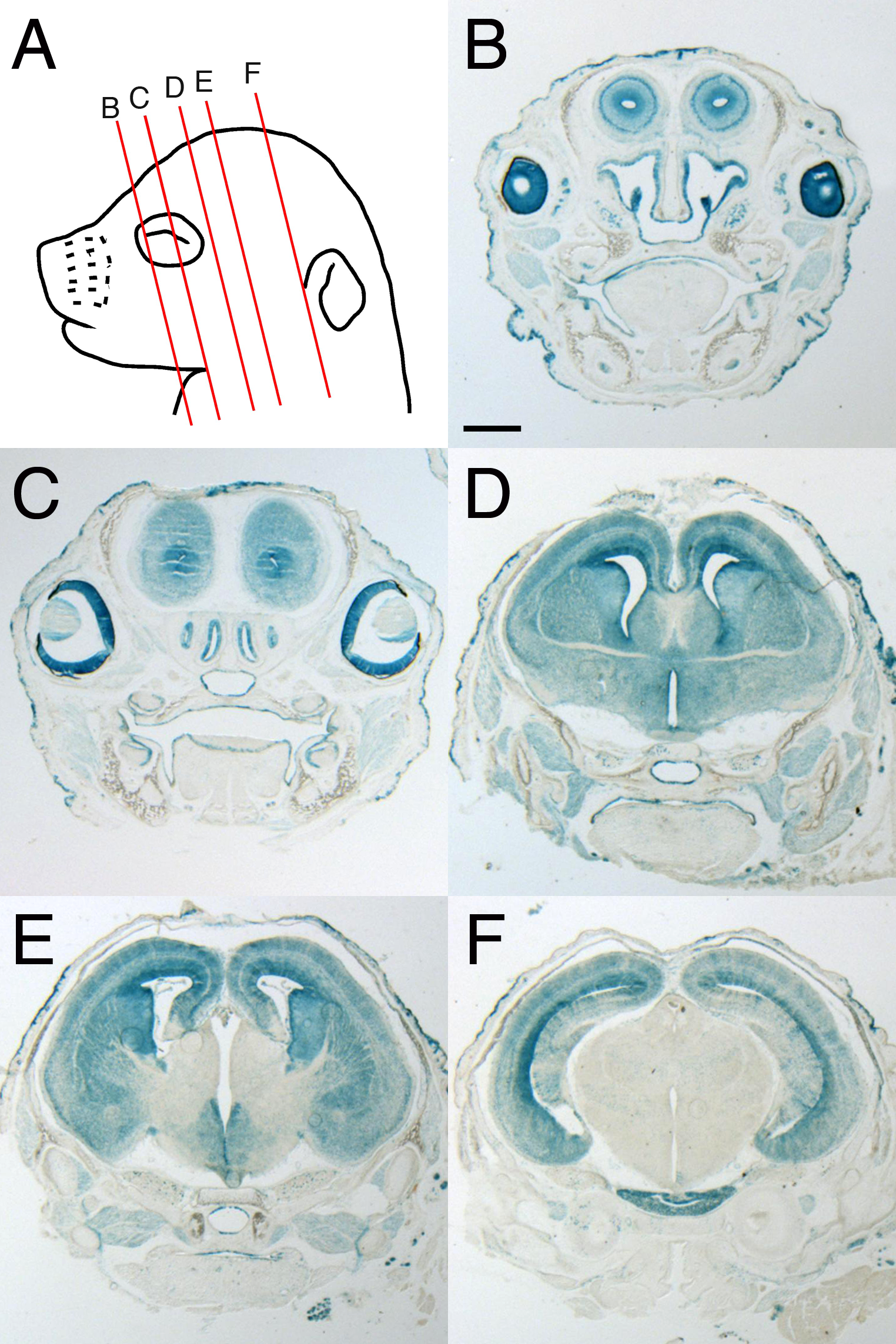

Supplement: Figure S1 — Cre-expression pattern in Rx-cre mice. Serial coronal sections through E17.5 heads of Rx-cre; R26R-lacZ mice after x-gal staining. The blue signal indicates expression of cre. (A) Schematic showing the level of sections along the anterior-posterior axis depicted in the other panels. (B–F) Cre expression is strongest in the retina (B,C) but also visible in more broadly in structures within the forebrain. (4.49 MB TIF) [file pone.0010021.s001.tif]

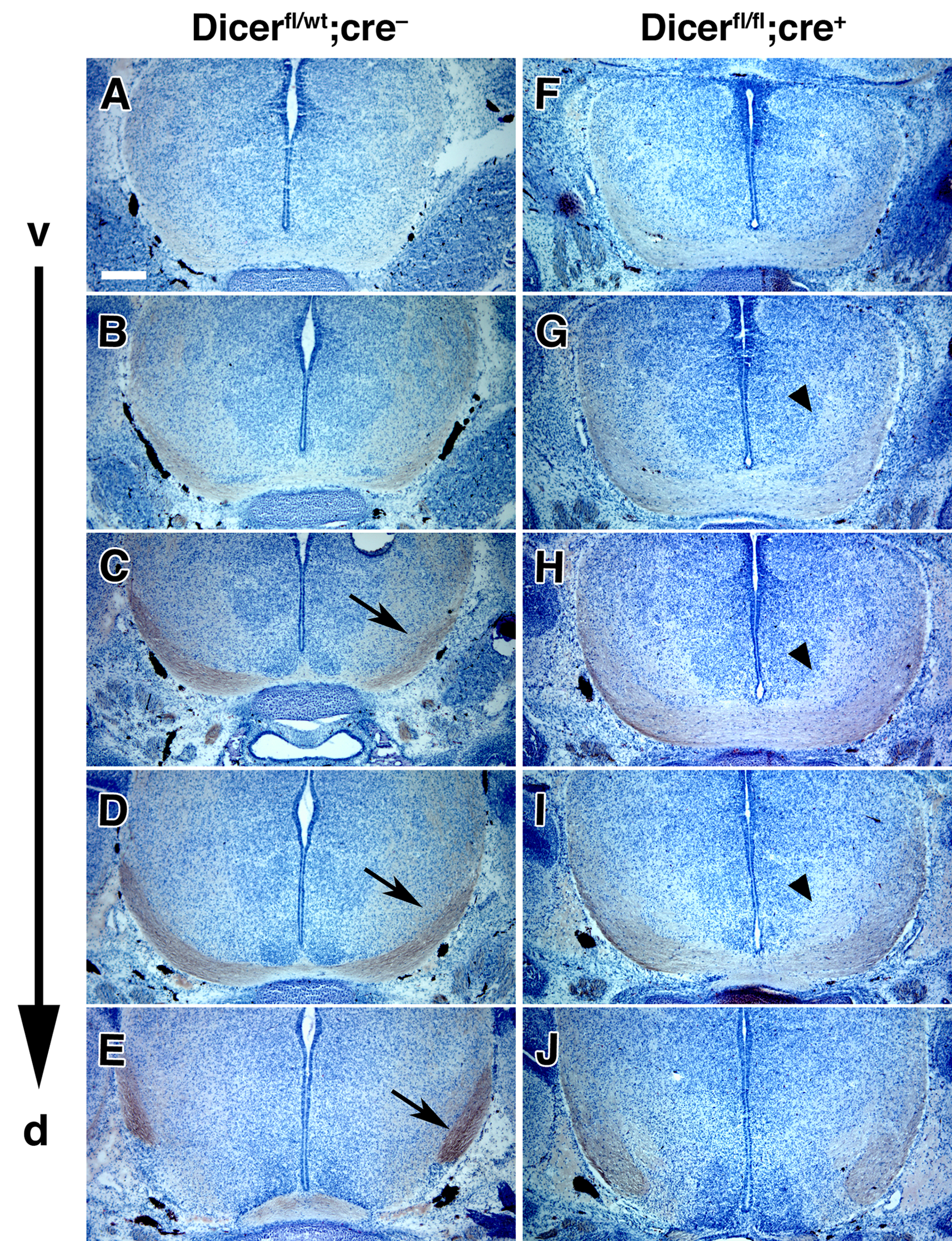

Supplement: Figure S2 — Disorganization of retinal fibers at the midline in Dicer mutant mice. Horizontal sections through the optic chiasm of E17.5 wild-type (A–E) and Dicer mutant (F–J) embryos, followed by immunohistochemistry using L1 antibody (brown staining) and counterstaining with haematoxylin (blue). (A–E) In wild-type embryos the retinal fiber bundles are crossing at the chiasm and extend further around the ventral diencephalon (arrows), leading to strong L1 staining. (F–J) In Dicer mutant embryos, L1 staining is much weaker across the entire chiasm, possibly because axons have defasciculated. L1-positive fibres can be found not only at the surface of the diencephalon, but also deeper in the tissue (arrowheads). v, ventral; d, dorsal. Scale bar = 200 µm. (10.02 MB TIF) [file pone.0010021.s002.tif]
